# Supplementary material for: Attenuation of clinical and immunological outcomes during SARS‐CoV‐2 infection by ivermectin
Source: EMBO Mol Med. 2021 Jul 12;13(8):e14122. doi: 10.15252/emmm.202114122 (PMC8350903; doi:10.15252/emmm.202114122)
Supplement: Supplementary file 1 — Appendix [file EMMM-13-e14122-s005.docx]

**Attenuation of clinical and immunological outcomes during SARS-CoV-2 infection by ivermectin**

Guilherme Dias de Melo^1^, Françoise Lazarini^2^, Florence Larrous^1^, Lena Feige^1^, Etienne Kornobis^3,4^, Sylvain Levallois^5^, Agnès Marchio^6^, Lauriane Kergoat^1^, David Hardy^7^, Thomas Cokelaer^3,4^, Pascal Pineau^6^, Marc Lecuit^5,8^, Pierre-Marie Lledo^2^, Jean-Pierre Changeux^9^, Hervé Bourhy^1*^

**Appendix**

Table of contents

Figures

- Appendix Figure S1. Virologic aspects in the nasal turbinates and in the lungs at 4 days post-infection of SARS-CoV-2-infected hamsters with and without ivermectin treatment.

Tables

- Appendix Table S1. Primer sequences used for qPCR in the golden hamster tissues.
- Appendix Table S2. Primer and probes sequences used for ddPCR in the golden hamster lung.

References

**
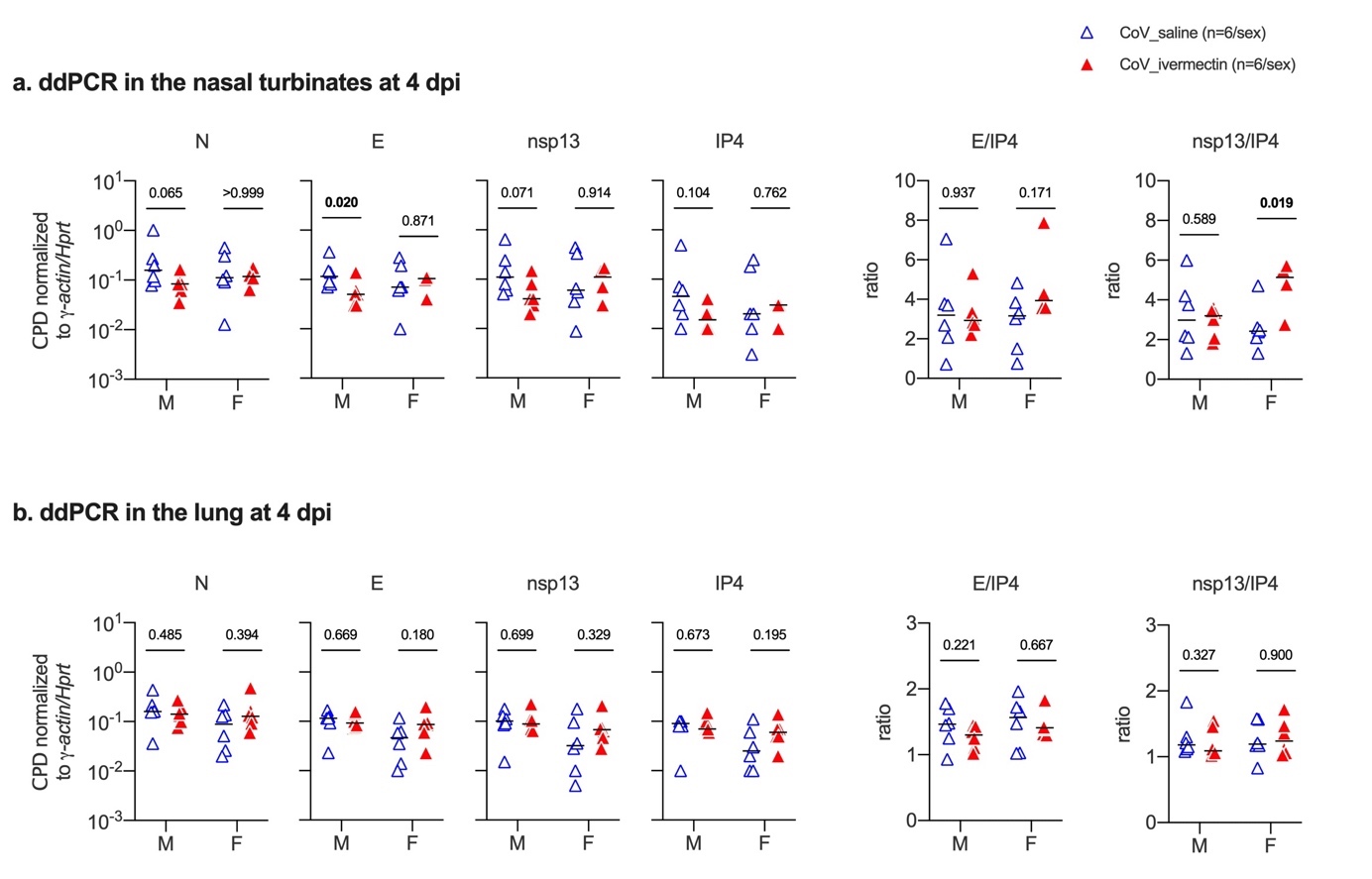
**

**Appendix Figure S1 | Virologic aspects in the nasal turbinates and in the lungs at 4 days post-infection of SARS-CoV-2-infected hamsters with and without ivermectin treatment. ab,** viral gene expression of N (nucleocapsid), E (envelope), nsp13 (non-structural protein 13), IP4 (RdRp, RNA-dependent RNA polymerase), and E/IP4 and nsp13/IP4 ratios determined by digital droplet PCR (ddPCR) in the nasal turbinates (a) and in the lungs (b) at 4 dpi. Data are expressed as CPD (copy per droplet) normalized to *γ−actin* and *Hprt* reference genes relative expression. n=6/group (except nasal turbinates from female CoV_ivermectin, where n=4). The p value is indicated in bold when significant at a 0.05 threshold. Mann-Whitney test. Horizontal lines indicate the medians. M: male hamsters; F: female hamsters. Data were obtained from two independent experiments for each sex.

**Appendix Table S1 | Primer sequences used for qPCR in the golden hamster tissues.**

| **Gene** | **Primer sequence (5' – 3')** | | **Reference** |
| --- | --- | --- | --- |
| ha-γ-*actin* | For | ACAGAGAGAAGATGACGCAGATAAT | (Ribeiro-Romão et al., 2016) |
|  | Rev | GCCTGAATGGCCACGTACA |  |
| ha-*Hprt* | For | TGCGGATGATATCTCAACTTTAACTG | (Zivcec et al., 2011) |
|  | Rev | AAAGGAAAGCAAAGTTTGTATTGTCA |  |
| ha-*actinB* | For | GGCCAGGTCATCACCATT | (Boudewijns et al., 2020) |
|  | Rev | GAGTTGAATGTAGTTTCGTGGATG |  |
| ha-*Il-6* | For | GGACAATGACTATGTGTTGTTAGAA | (Ribeiro-Romão et al., 2016) |
|  | Rev | AGG CAA ATT TCC CAA TTG TAT CCA |  |
| ha-*Il-10* | For | GGTTGCCAAACCTTATCAGAAATG | (Ribeiro-Romão et al., 2016) |
|  | Rev | TTCACCTGTTCCACAGCCTTG |  |
| ha-*Il-1β* | For | GGCTGATGCTCCCATTCG | (Zivcec et al., 2011) |
|  | Rev | CACGAGGCATTTCTGTTGTTCA |  |
| ha-*Tnf-α* | For | TGAGCCATCGTGCCAATG | (Ribeiro-Romão et al., 2016) |
|  | Rev | AGCCCGTCTGCTGGTATCAC |  |
| ha-*Ifn-β* | For | ACCCTAAAGGAAGTGCCAG | (Gowen et al., 2015) |
|  | Rev | CCAGCTGCCAGTAATAGCTC |  |
| ha-*Ifn-γ* | For | TGTTGCTCTGCCTCACTCAGG | (Ribeiro-Romão et al., 2016) |
|  | Rev | AAGACGAGGTCCCCTCCATTC |  |
| ha-*Cxcl10* | For | GCCATTCATCCACAGTTGACA | (Zivcec et al., 2011) |
|  | Rev | CATGGTGCTGACAGTGGAGTCT |  |
| ha-*Ccl5* | For | ACTGCCTCGTGTTCACATCA | (Schountz et al., 2019) |
|  | Rev | CCCACTTCTTCTTTGGGTTG |  |
| ha-*Tnfaip3* | For | TGGAAAGCCAGAAGAAGCTC | ENSMAUG00000019454^§^ |
|  | Rev | ATGAGGCAGTTTCCATCACC |  |
| ha-*Casp3* | For | AAGCCATGGTGATGAAGGAG | ENSMAUG00000004837 |
|  | Rev | TCATGTCATCTTCGGTTCCA |  |
| ha-*Plcb1* | For | ACCCATGGATTCACCATGAC | ENSMAUG00000015228 |
|  | Rev | GAATGCACACTCTGCAATGG |  |
| ha-*Chrna7* | For | TCCCTCCAGGCATATTCAAG | ENSMAUG00000005544 |
|  | Rev | TCCCATGAGATCCCACTCTC |  |
| ha-*Chrnb4* | For | CTGAACAAAACCCGCTACAAC | ENSMAUG00000012409 |
|  | Rev | TCTCGCTCATTCACACTGATG |  |
| ha-*Adra1d* | For | TCTTCGTCCTTTGCTGGTTC | ENSMAUG00000009086 |
|  | Rev | GCCAGAAGATGACCTTGAAGAC |  |
| ha-*Grind2* | For | AAATCCATCTGGCTGCTCTG | ENSMAUG00000015271 |
|  | Rev | GAAAGATGACGGCGAAGAAG |  |
| ha-*Pik3r5* | For | CTGCAAACCCTGGAAAACTC | ENSMAUG00000020086 |
|  | Rev | TGTCAAAGCTGTCCTGGTTC |  |
| ha-*Igf1r* | For | ATCAGAAAGTACGCGGATGG | ENSMAUG00000010750 |
|  | Rev | CTTCTCGGCTTCAGTTTTGG |  |
| ha- *Ifn-λ* | For | CCCACCAGATGCAAAGGATT | (Boudewijns et al., 2020) |
|  | Rev | CTTGAGCAGCCACTCTTCTATG |  |
| ha- *Mx2* | For | CCAGTAATGTGGACATTGCC | (Ribeiro-Romão et al., 2016) |
|  | Rev | CATCAACGACCTTGTCTTCAGTA |  |
| ha-*Wnt11* | For | TACGCATGAGTTCCAGGTTG | ENSMAUG00000008427 |
|  | Rev | TGTGCTATGGCATCAAGTGG |  |
| ha-*Sfrp4* | For | TGTTACGAGTGGCGATCAAG | ENSMAUG00000018868 |
|  | Rev | TTGTGGGGTTTTCTGTGCAG |  |
| ha-*Epha2* | For | ACAAGGCAGAAGGTGATTGG | ENSMAUG00000009598 |
|  | Rev | CAGCTTTCAGCGTCTTGATG |  |
| ha-*Hgf* | For | GCTGGAGCTGAAAAGATTGG | ENSMAUG00000007722 |
|  | Rev | TCGGACGAAAATACCAGGAC |  |
| ha-*Il-2* | For | CATCTTCCAAGTGAAAGCTTTTGCT | (Ribeiro-Romão et al., 2016) |
|  | Rev | CCAGTGCCTGGAAGAAGAACTT |  |
| ha-*Il-2ra* | For | AAAGCAAGCTACACCTAACCC | (Ribeiro-Romão et al., 2016) |
|  | Rev | GCCTTGTATCCTTGAATGCG |  |
| ha-*Tgf-β* | For | GGCTACCACGCCAACTTCTG | (Ribeiro-Romão et al., 2016) |
|  | Rev | GAGGGCAAGGACCTTACTGTACTG |  |
| ha-*Gabbr1* | For | AAAGATCAACGACCACAGGG | ENSDARG00000016667 |
|  | Rev | GGTGACAGGAGCAGTGATAAGAG |  |
| ha-*Prkg1* | For | ATCGAGGTTATGCCAAGCTG | ENSMAUG00000009610 |
|  | Rev | TGGAGTCCCACAAAAAGTCC |  |
| ha-*Gnai1* | For | ACCAGGGTGAAAACAACAGG | ENSMAUG00000000903 |
|  | Rev | CCCTTCAAAGCAGTGAATCC |  |
| ha-*Grid1* | For | TGGCTTCTTCCCTGGATATG | ENSMAUG00000003072 |
|  | Rev | TGTCATCATTGAGGCTCAGG |  |
| ha-*Krt4* | For | ACATCAATGCCCTGAGGAAG | ENSMAUG00000018774 |
|  | Rev | CAGCTGTGCGCTTGTTAATC |  |
| ha-*Wnt3a* | For | AATGGTGTCTCGGGAGTTTG | ENSMAUG00000021475 |
|  | Rev | GTTGTTGTGACGGTTCATGG |  |
| ha-*Creb5* | For | ATCAGAAAGTACGCGGATGG | ENSMAUG00000008771 |
|  | Rev | CTTCTCGGCTTCAGTTTTGG |  |
| ha-*Ager* | For | CCCTCGAACACAGTCTCCAT | ENSMAUG00000001006 |
|  | Rev | CACAGCTGTAGATGCCCTCA |  |
| ha-*Fos* | For | AATGGTGAAGACCGTGTCAG | ENSMAUG00000019419 |
|  | Rev | TCCTTTCCCTTCGGATTCTC |  |
| ^§^ Designed by this paper, based on the Ensembl gene number, available at <www.ensembl.org> | | | |

**Appendix Table S2 | Primer and probes sequences used for ddPCR in the golden hamster lung.**

| **Gene** | **Primer sequence (5' – 3')** | | **Concentration**  **(nM)** | **Reference** |
| --- | --- | --- | --- | --- |
| N | For | GGGGAACTTCTCCTGCTA | 900 | (Suo et al., 2020) |
|  | Rev | CAGACATTTTGCTCTCAA | 900 |  |
|  | Probe | FAM-TTGCTGCTGCTTGACAGATT-IBFQ | 250 |  |
| IP4 | For | GGTAACTGGTATGATTTCG | 900 | (WHO, 2020) |
|  | Rev | CTGGTCAAGGTTAATATAGG | 900 |  |
|  | Probe | HEX-TCATACAAACCACGCCAGG-IBFQ | 300 |  |
| E | For | ACAGGTACGTTAATAGTTAATAGCGT | 200 | (Corman et al., 2020) |
|  | Rev | ATATTGCAGCAGTACGCACACA | 200 |  |
|  | Probe | FAM-ACACTAGCCATCCTTACTGCGCTTCG-IBFQ | 150 |  |
| nsp13 | For | TAAGGGCACACTAGAACCAG | 900 | this paper |
|  | Rev | ACAATTTCAGCAGGACAACG | 900 |  |
|  | Probe | HEX-AGGTCCAGACATGTTCCTCGGAA-IBFQ | 250 |  |

**REFERENCES**

Boudewijns R, Thibaut HJ, Kaptein SJF, Li R, Vergote V, Seldeslachts L, Van Weyenbergh J, De Keyzer C, Bervoets L, Sharma S et al. (2020) STAT2 signaling restricts viral dissemination but drives severe pneumonia in SARS-CoV-2 infected hamsters. Nature Commun 11: 5838-5838

Corman VM, Landt O, Kaiser M, Molenkamp R, Meijer A, Chu DK, Bleicker T, Brünink S, Schneider J, Schmidt ML et al. (2020) Detection of 2019 novel coronavirus (2019-nCoV) by real-time RT-PCR. Euro Surveill 25: 2000045

Gowen BB, Westover JB, Sefing EJ, Bailey KW, Nishiyama S, Wandersee L, Scharton D, Jung K-H, Ikegami T (2015) MP-12 virus containing the clone 13 deletion in the NSs gene prevents lethal disease when administered after Rift Valley fever virus infection in hamsters. Front Microbiol 6

Ribeiro-Romão RP, Saavedra AF, Da-Cruz AM, Pinto EF, Moreira OC (2016) Development of real-time PCR assays for evaluation of immune response and parasite load in golden hamster (Mesocricetus auratus) infected by Leishmania (Viannia) braziliensis. Parasit Vectors 9: 361-361

Schountz T, Campbell C, Wagner K, Rovnak J, Martellaro C, DeBuysscher BL, Feldmann H, Prescott J (2019) Differential Innate Immune Responses Elicited by Nipah Virus and Cedar Virus Correlate with Disparate In Vivo Pathogenesis in Hamsters. Viruses 11: 291

Suo T, Liu X, Feng J, Guo M, Hu W, Guo D, Ullah H, Yang Y, Zhang Q, Wang X et al. (2020) ddPCR: a more accurate tool for SARS-CoV-2 detection in low viral load specimens. Emerging Microbes & Infections 9: 1259-1268

WHO (2020) Protocol: Real-time RT-PCR assays for the detection of SARS-CoV-2. Institut Pasteur, Paris. <https://www.who.int/docs/default-source/coronaviruse/real-time-rt-pcr-assays-for-the-detection-of-sars-cov-2-institut-pasteur-paris.pdf?sfvrsn=3662fcb6_2>

Zivcec M, Safronetz D, Haddock E, Feldmann H, Ebihara H (2011) Validation of assays to monitor immune responses in the Syrian golden hamster (Mesocricetus auratus). J Immunol Methods 368: 24-35
