# Supplementary material for: Attenuation of clinical and immunological outcomes during SARS‐CoV‐2 infection by ivermectin
Source: EMBO Mol Med. 2021 Jul 12;13(8):e14122. doi: 10.15252/emmm.202114122 (PMC8350903; doi:10.15252/emmm.202114122)
Supplement: Supplementary file 2 — Expanded View Figures PDF [file EMMM-13-e14122-s002.pdf]

Expanded View Figures

Figure EV1. Complementary clinical aspects and olfaction test in hamsters, infected or not by the SARS-CoV-2, with and without ivermectin treatment.

A Progression of body weight in male and female hamsters, mock-infected or SARS-CoV-2-infected, treated with saline or with 400 µg/kg ivermectin. Symbols indicate the median ± interquartile range.
B Curves represent the percentage of animals that did not find the hidden (buried) food.
C Curves represent the percentage of animals that did not find the visible (unburied) food.
D Summary of olfactory status.

Data information: Food finding assays were performed at 3 days post-infection. $n = 6$/group, except males CoV_saline and males CoV_ivermectin, where $n = 12$/group. The *P* value is indicated in bold when significant at a 0.05 threshold. Log-rank (Mantel–Cox) test (B, C) and Fisher's exact test (D). Data were obtained from three independent experiments for males and two independent experiments for females. The data for CoV_saline and CoV_ivermectin groups (B) are already presented in Fig 1B.
Source data are available online for this figure.

© 2021 The Authors

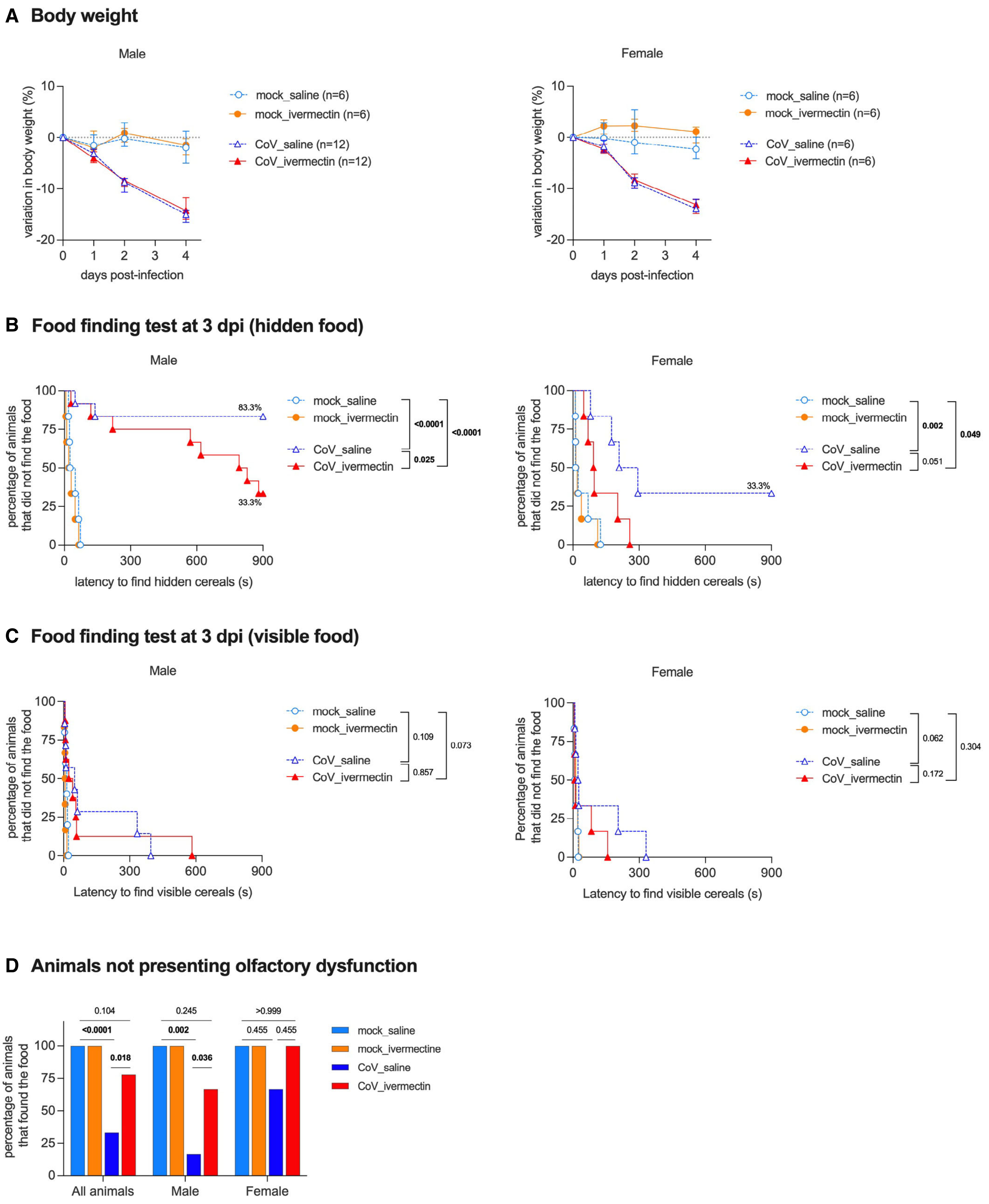

Figure EV1.

© 2021 The Authors

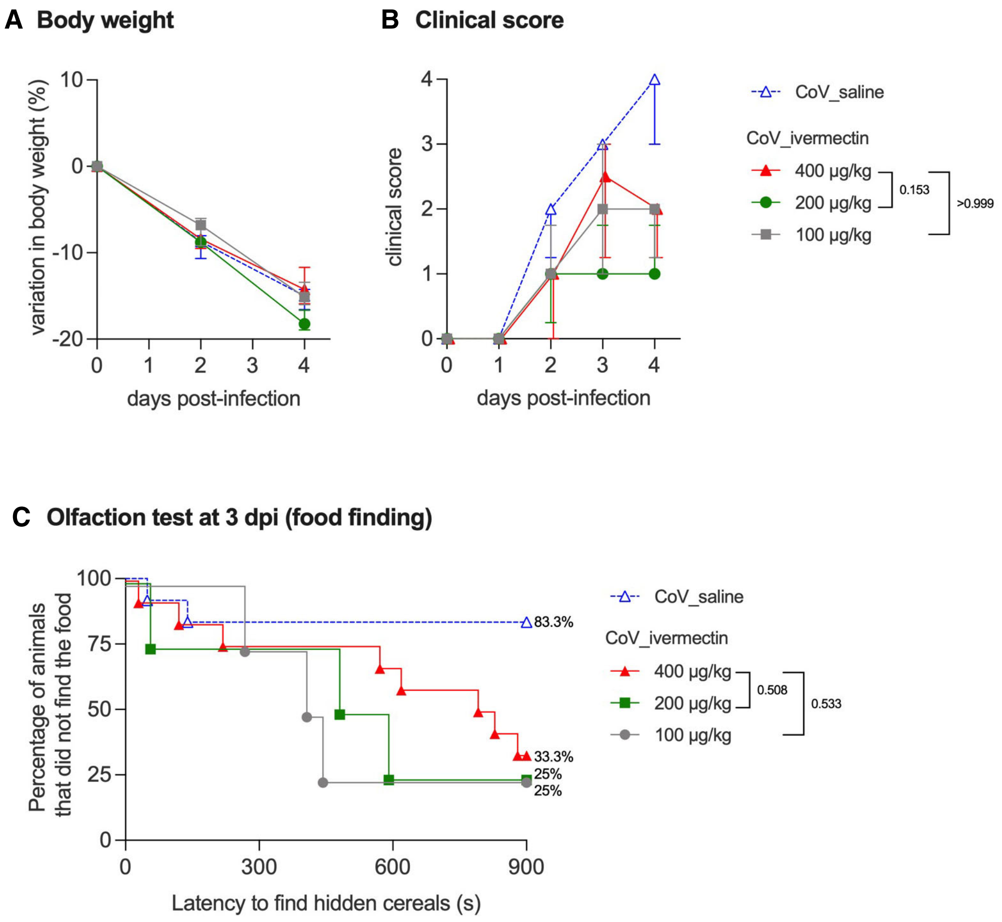

Figure EV2. Clinical aspects of SARS-CoV-2-infected male hamsters and treated with different doses of ivermectin.

A Progression of body weight in male hamsters, treated with saline or with 400, 200, or 100 µg/kg ivermectin. Symbols indicate the median ± interquartile range.
B Clinical score based on a cumulative 0–4 scale: ruffled fur; slow movements; apathy; and absence of exploration activity. Symbols indicate the median ± interquartile range.
C Olfaction deficit based on the buried food finding test. Curves represent the percentage of animals that did not find the buried food. Food finding assays were performed at 3 days post-infection.

Data information: *n* = 12/group (CoV_saline and CoV_ivermectin 400 µg/kg, as shown in Fig 1) or *n* = 4 (CoV_ivermectin 200 µg/kg and 100 µg/kg). The *P* value is indicated in bold when significant at a 0.05 threshold. Mann–Whitney test at 4 dpi (B) and log-rank (Mantel–Cox) test (C). The data for CoV_saline and CoV_ivermectin 400 µg/kg groups (B, C) are already presented in Fig 1B.
Source data are available online for this figure.

Figure EV3. Transcriptomic aspects in the lung of SARS-CoV-2-infected hamsters compared to mock-infected hamsters at 4 days post-infection.

A KEGG enrichment.
B GO enrichment analysis.

Data information: Selected terms are based on the up- and downregulated genes between infected (CoV_saline) and mock-infected (mock_saline) samples. Only the 20 highest fold enrichments are plotted for the upregulated gene set. Circle sizes are proportional to the gene set size, which shows the total size of the gene set associated with GO terms. Circle color is proportional to the corrected *P*-values. Terms identified in both sexes are marked in bold. Complete analyses are listed in Dataset EV1.
Source data are available online for this figure.

© 2021 The Authors

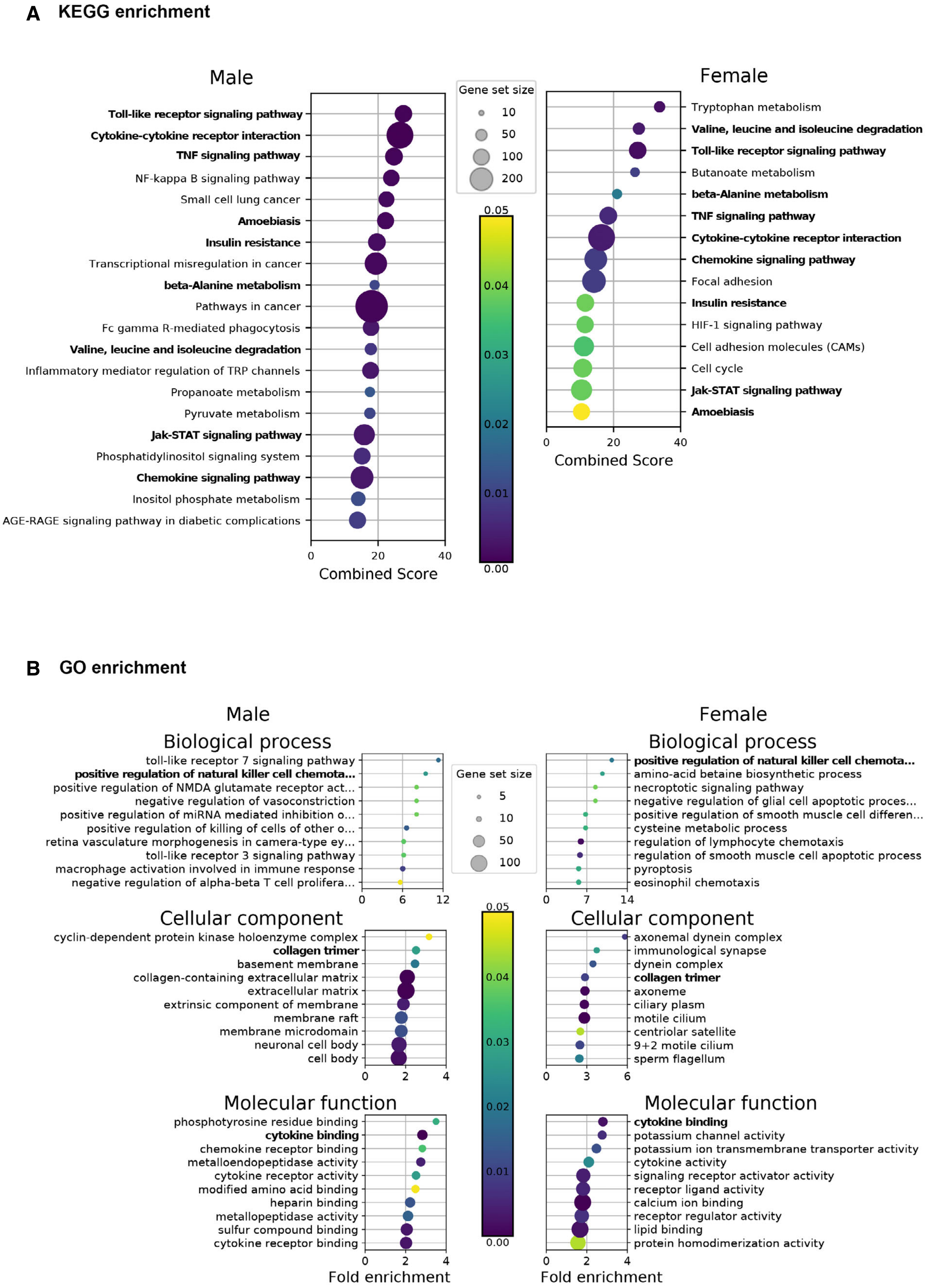

Figure EV3.

 © 2021 The Authors

Figure EV4. Transcriptomic aspects in the lung of SARS-CoV-2-infected and ivermectin-treated hamsters compared to SARS-CoV-2-infected and saline-treated hamsters at 4 days post-infection.

A KEGG enrichment.
B GO enrichment analysis.

Data information: Selected terms are based on the up- and downregulated genes between IVM-treated (CoV_ivermectin) and saline-treated (CoV_saline) samples. Only the 20 highest fold enrichments are plotted for the upregulated gene set. Circle sizes are proportional to the gene set size, which shows the total size of the gene set associated with GO terms. Circle color is proportional to the corrected *P*-values. Complete analyses are listed in Dataset EV1.
Source data are available online for this figure.

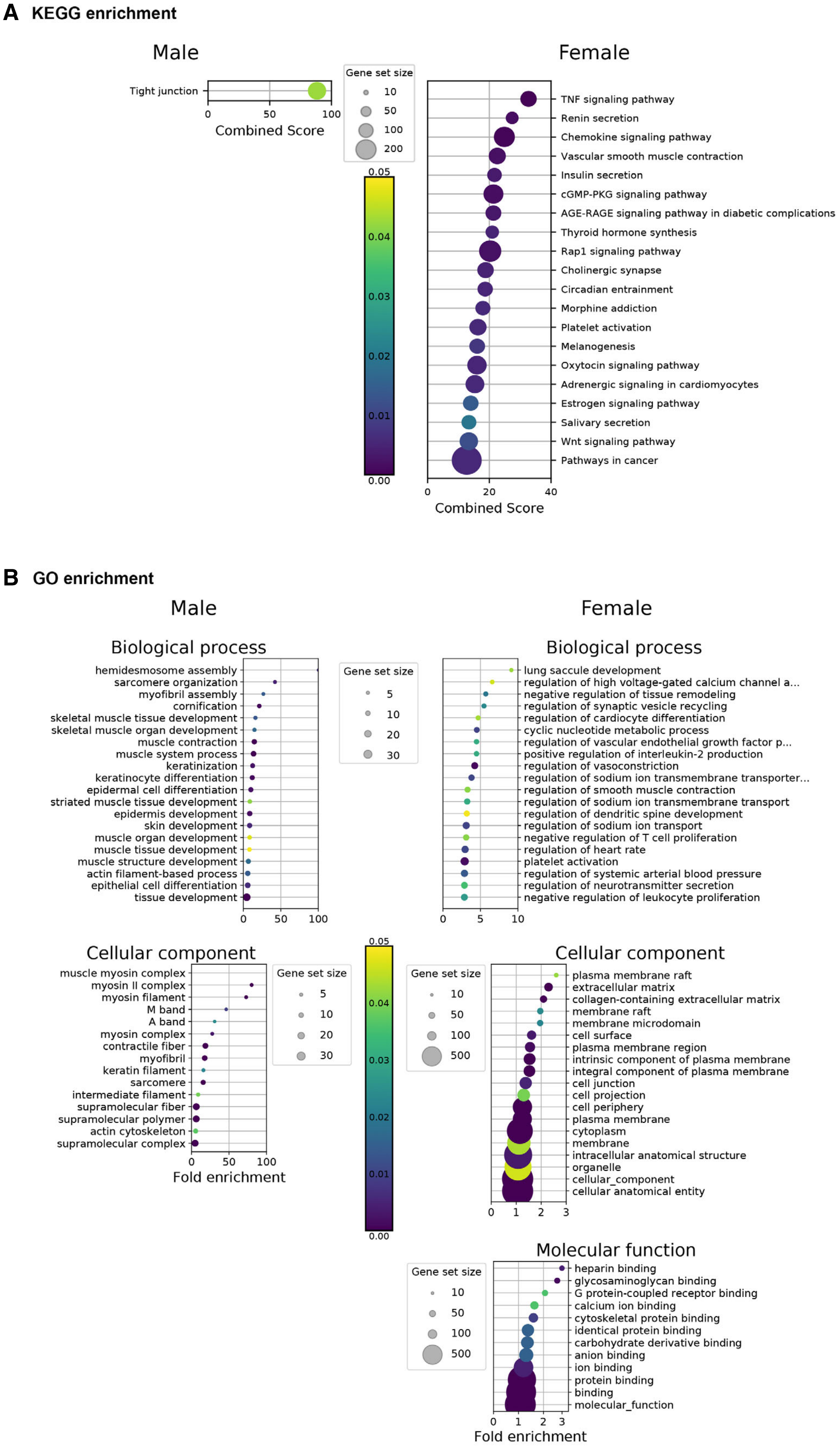

Figure EV4.

 © 2021 The Authors

Figure EV5. Transcriptomic profile in the lung of SARS-CoV-2-infected hamsters with and without ivermectin treatment at 4 days post-infection.

A Heatmaps showing the differentially expressed genes according to the selected KEGG pathways, calculated in comparison with mock-infected hamsters. * indicates Benjamini–Hochberg-adjusted *P*-value<0.05 in the comparison between saline and ivermectin within the same sex. Color gradient represents the transcription $\log_2$ fold change comparing infected and mock-infected. Complete analyses are listed in Dataset EV1.

B Validation targets in the lung at 4 dpi. Horizontal lines indicate medians. The *P* value is indicated in bold when significant at a 0.05 threshold. Mann–Whitney test.

Data information: M: male hamsters and F: female hamsters. Data were obtained from two independent experiments for each sex.
Source data are available online for this figure.

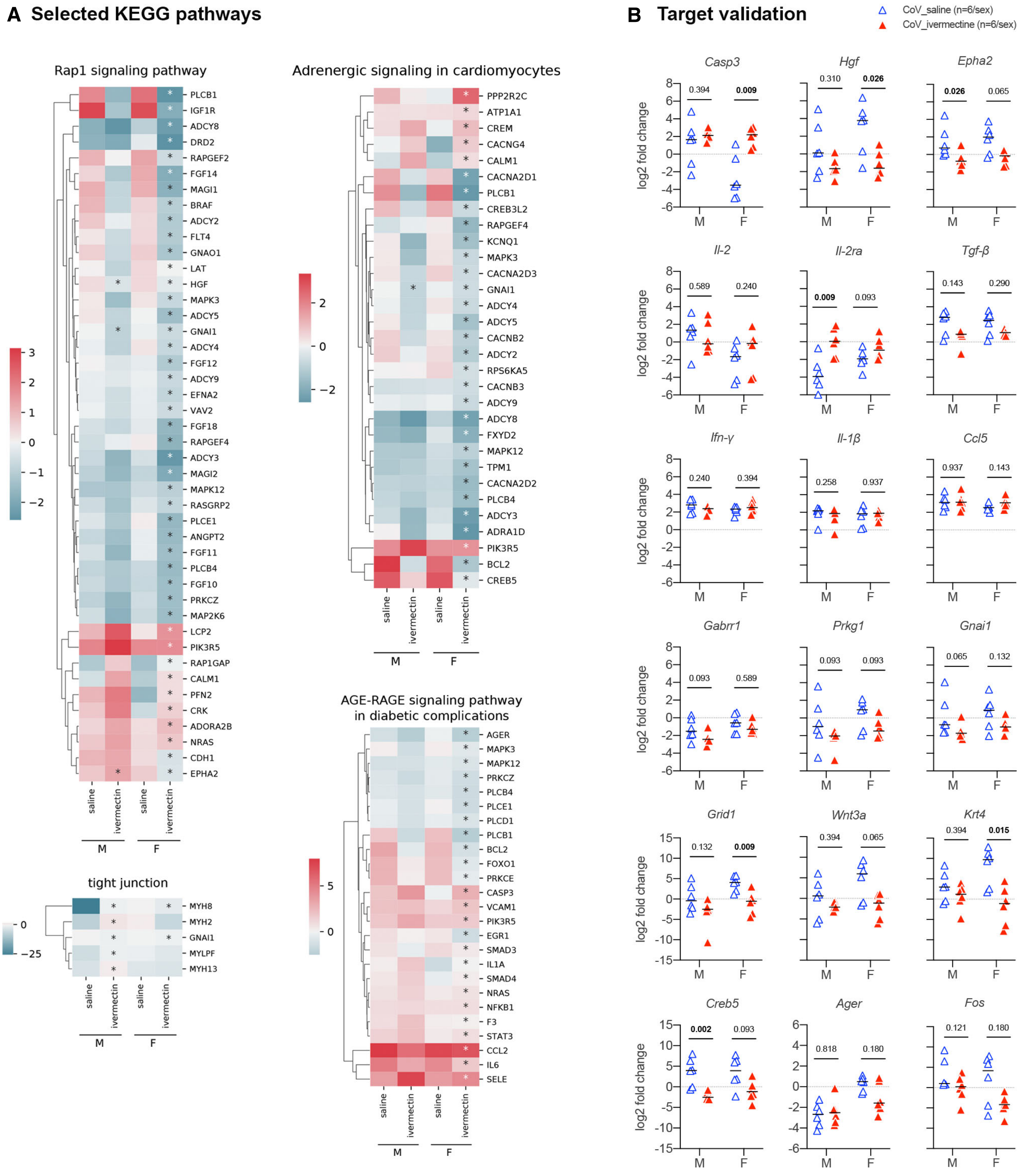

Figure EV5.

 © 2021 The Authors
